# Albuminuria in Phthisis

**Published:** 1907-11-30

**Authors:** 


### Clinical Points.

### ALBUMINURIA IN PHTHISIS.

It often happens that a patient suffering from phthisis is found to have albuminuria, and the question arises: What is its significance? One has a vague feeling that it may be due to the beginning of lardaceous change; that it may be due to the very low pressure in these cases; that it may be simply "febrile"; that it may be due to actual tubercles in the kidney; that it may be whatever "functional" means; or that it may indicate some lesion in the kidneys other than tubercle or lardaceous degeneration.

Analysis of a very large number of cases has established certain main points:—

1. Albuminuria in phthisis is seldom severe, but is very common in mild degree.
2. The incidence of albuminuria is very much greater than is that of lardaceous disease; so that albumen in the urine is no proof of the existence of the latter.
3. The kidneys in cases of fatal phthisis nearly always show the microscopic changes of subacute, or even acute, tubal or tubal and interstitial nephritis.
4. Nevertheless the amount of nephritis found in sections of the kidneys is chiefly of laboratory interest, for the clinical symptoms of nephritis are nearly always absent.
5. The albuminuria is therefore not "functional" in the sense that the kidneys are not affected at all; but to all intents and purposes it may be treated as though it were functional from a therapeutic point of view. It cannot be regarded as functional from a prognostic standpoint, however.
6. Males with phthisis are slightly more apt to have albuminuria than are females with phthisis.
7. There is no correlation between the existence of albuminuria and of œdema; the urine may contain albumen when there is no œdema, and *vice versa*.
8. The longer the phthisis has existed up to about 10 years the more likely is albumen to be present; those who have survived phthisis for longer than ten years are a little less likely to have albuminuria than are those who have reacted against the disease less successfully.
9. Renal tube-casts are present in the centrifugalised deposits from the urine in many cases of phthisis with albuminuria; this confirms the histological findings in the kidneys in regard to the presence of some actual nephritis, but it does not prove that there is enough nephritis to be itself dangerous. The laboratory report will nearly always show the presence of both granular and hyaline casts in these cases. It is important to know their significance here. They by no means indicate any necessity to treat the patients on nephritic lines, and they do not prove lardaceous degeneration to be present.
10. The effect of age upon the likelihood of albuminuria being present is well marked; it is least likely to occur in middle-aged patients, and most likely in those who are quite young or quite elderly.
11. The existence of albuminuria in phthisis is no proof that the patient is bound to die soon. It is possible for a consumptive patient to be cured and to cease passing albumen in the urine; and it is also possible for a patient to survive for 20 years or more though passing albumen all the time. Nevertheless, the mortality among those who have albuminuria is very much greater than it is amongst those who have it not.

It may be said that the above conclusions are merely common sense; this may be so, but nevertheless common sense is sometimes wrong, and it is good to have actual facts upon which to confirm it. The following are some of the figures based upon examination of many hundreds of consecutive cases at the Phipps Institute for Consumption. The patients were in various stages of phthisis; some were out-patients, but the great majority were in-patients whose degree of illness varied from moderate to severe.

The albumen was tested for in the ordinary clinical way by heat and acetic acid, with control for nucleoproteid. Out of over 1,000 cases, 31 per cent. had albuminuria.

The following figures show clearly how much more liable the young and the elderly are to it than those whose ages are intermediate:—

| | Percentage of phthisis cases having albuminuria. |
|---|---|
| Under 10 years of age ... ... ... ... | 50 |
| Between 10 and 20 years of age ... ... | 27 |
| " 20 and 30 " " ... ... | 27 |
| " 30 and 40 " " ... ... | 35 |
| " 40 and 50 " " ... ... | 24 |
| " 50 and 60 " " ... ... | 49 |

Males had albuminuria in 33 per cent. of cases.
Females " " 28 " " "

Œdema is not common in phthisis; it was noticed in no more than 16 per cent. of all the cases, and even in those in which it occurred it was but slight.

Œdema was present in 19 per cent. of the albuminuria cases.
Œdema was present in 15 per cent. of non-albuminuria cases.

That is to say, there is no correlation between the two.

| When the phthisis had existed— | The percentage having albuminuria was:— |
|---|---|
| Less than 2 years ... ... ... ... | 26 |
| Between 2 and 5 years ... ... ... | 34 |
| " 5 and 10 " ... ... ... | 36 |
| " 10 and 20 " ... ... ... | 33 |
| Over 20 years ... ... ... ... ... | 20 |

It is not every patient who dies of phthisis who has had a clinical degree of albuminuria. It is possible for the end to come without albuminuria having been found at all. Nevertheless, the proportion of cases in which albuminuria occurred at the Phipps Institute was nearly three times as great among the fatal as among the non-fatal cases. Expressed in another way, in 116 cases of phthisis with albuminuria, 27 (or 23 per cent.) died during the period of observation; while in the 263 cases of non-albuminuria, only 10 (or 4 per cent.) died in the same period.

This, and the fact that slight albuminuria with renal tube-casts is no proof of lardaceous disease, are perhaps the two most practical points that the analyses of the figures teach.

